# Technology-Assisted Interventions for Reducing Risk of Suicide: A Meta-Analysis Focused on Suicidal Ideation

**DOI:** 10.62641/aep.v54i2.2089

**Published:** 2026-04-15

**Authors:** Martina Medolla, Fabio Corona, Ana Huertes-del Arco, Francisco Pablo Holgado-Tello, Miguel Á. Carrasco

**Affiliations:** ^1^Department of Personality, Assessment and Psychological Treatments, Faculty of Psychology, National University of Distance Education (UNED), 28040 Madrid, Spain; ^2^Faculty of Psychology and Health Sciences, Open University of Madrid (UDIMA), 28400 Madrid, Spain; ^3^Department of Economics and Statistical Science, University of Cagliari, 09123 Cagliari, Italy; ^4^Department of Methodology of Behavioral Sciences, Universidad Nacional de Educación a Distancia (UNED), 28040 Madrid, Spain

**Keywords:** suicide prevention, suicide intervention, adolescents, digital devices, technology

## Abstract

**Background:**

Suicide is a leading cause of death among adolescents worldwide. Suicide is a complex multifactorial issue and, in 2025, became the third-leading cause of death among individuals aged 15–29. We sought to evaluate the effectiveness of technology-assisted interventions (TIs) in reducing both suicidal behaviour and non-suicidal self-injury among adolescents.

**Methods:**

For this meta-analysis, we searched the EBSCO (APA PsycArticles, APA PsycInfo, MEDLINE, APA PsycTherapy, Psychology and Behavioral Sciences Collection), PubMed and Cochrane databases from inception until May 2025, seeking out articles featuring data (quantitative outcomes related to suicidal ideation or behaviour) on evaluated suicide or self-harm interventions among children and adolescents (aged 13–18) that incorporated digital technologies in some manner. We used random effects meta-analysis to estimate the effect size for suicidal ideation reduction. We assessed heterogeneity using the I^2^ statistic, and, due to the small number of considered studies, publication bias was assessed using an adaptation of Cochrane’s guidelines for the assessment of bias risk. The review was registered with INPLASY, with the code INPLASY202570073.

**Results:**

After applying the eligibility criteria, six studies were selected for the analysis. Although the initial conceptual aim pertained to suicidal risk and self-harm more broadly, suicidal ideation was the only outcome consistently reported across the eligible studies; therefore, it served as the primary meta-analytic outcome.

**Conclusions:**

The results highlight that technology-assisted interventions yield an overall statistically significant moderate reduction in suicidal ideation, providing valuable support for the implementation of such interventions during adolescence, although further rigorous research is needed to strengthen the evidence base.

## Introduction 

Suicide is a leading cause of death among adolescents worldwide. According to the World Health Organization [1], more than 720,000 people die by suicide each year. Suicide is a complex multifactorial issue and, in 2025, became the third-leading cause of death among individuals aged 15–29 [1].

Adolescence is a developmental period characterised by profound biological, cognitive and socio-emotional transitions, potentially increasing vulnerability to psychological distress and suicidal outcomes [1, 2, 3]. Digital technologies play an integral role in adolescents’ daily lives [4]. This pervasive engagement has positioned digital tools as promising avenues for youth suicide prevention. Recent evidence suggests that adolescents exhibit strong adherence to digital interventions, which may contribute to better suicide-related outcomes [5].

Technology-assisted interventions may be situated within the broader framework of behavioural intervention technologies (BITs) [6], a class of psychosocial and therapeutic approaches that deliver core intervention components through digital tools. As described by Mohr* et al*. [6], BITs can employ a wide range of media, “including but not limited to telephone and videoconferencing, web-based (internet) interventions, mobile-device-based (mHealth) interventions, sensor-based patient monitoring, social media, virtual reality, and gaming” (p. 333). A comprehensive understanding of suicidal behaviour—from early suicidal ideation to planning, attempts and, eventually, suicide—is essential to guiding prevention efforts [7, 8]. Digital technologies are particularly well positioned to support early and responsive interventions along this trajectory.

Evidence highlights the importance of identifying self-injurious behaviour early. A recent umbrella review identified a history of self-injury as one of the strongest predictors of suicidal ideation and behaviours among adolescents [9]. A cross-sectional study also found a consistent significant association between non-suicidal self-injury (NSSI) and suicidal behaviour (χ^2^ = 58.16, *p* < 0.001) [10]. Evidently, detecting and addressing self-injurious behaviour must constitute an important element in the design of youth suicide-prevention strategies.

This is not only a clinical priority but also an opportunity for innovation. Contemporary adolescents are the most digitally connected generation in history [11], with the vast majority using digital devices daily and many reporting near-constant online engagement [12]. Digital environments play a central role in shaping their experiences [13], making technology-assisted interventions (TIs) a particularly relevant and promising avenue for suicide prevention in this age group [14].

This meta-analysis examines the application and effectiveness of technology-assisted suicide-prevention interventions specifically for adolescents. As highlighted by Gaynor *et al*. [15], there is a clear need for reviews centred on individuals under 18, with most existing syntheses combining adolescents and young adults aged 12–25 [16]. Only a limited number of reviews have concentrated exclusively on adolescents aged 12–18 [17], underscoring the relevance and novelty of the current study.

To our knowledge, no previous meta-analysis has focused exclusively on TIs targeting suicidal ideation among adolescents, making this a distinct contribution to the broader literature on digital suicide prevention. Through this integrated systematic review and meta-analysis, we aimed to examine the following: (1) the effects of previous technology-assisted interventions on suicidal behavior in adolescents; (2) the impact of incorporating digital technologies into those interventions; and (3) the relative influence of different variables behind suicidal behaviors among adolescents, including the nature of intervention exposure (i.e., type of control condition) and the type of technology used.

## Methods

### Search Strategy and Selection Criteria

We conducted a systematic literature review, following Preferred Reporting Items for Systematic reviews and Meta-Analyses (PRISMA) [18] guidelines, to identify TIs for reducing both suicidal and self-injury ideation among adolescents. The review was registered with INPLASY, with the code INPLASY202570073.

The following databases were utilised for the research: EBSCO (APA PsycArticles, APA PsycInfo, MEDLINE, APA PsycTherapy, Psychology and Behavioral Sciences Collection), PubMed and Cochrane. No language restrictions were applied. The research covered the period from the date on which each database was created to 15 May 2025. None of the databases applied predetermined temporal restrictions that could have limited the retrieval of eligible records. Grey literature (e.g., theses, technical reports, non-indexed conference proceedings, institutional documents) was not included, as the aim of this review was to focus exclusively on peer-reviewed studies available in the considered academic databases.

The same research query was used and adapted for the syntax of each database: (1) (Intervention OR Treatment OR psychotherapy OR Counsel*ing OR Psycho* treatment OR Psycho*treatment* OR Psycho* intervention* OR Psycho* therap* OR Psycho*therap* OR Supportive therap* OR Supportive treatment*) AND (2) (Suicid* OR Suicidal Behavior OR Youth Suicide) AND (3) (Adolescents OR Teenagers) AND (4) (Digital devices OR Smart devices OR Tablets OR social media OR Technology OR Internet OR Smartphone).

Some selection criteria were employed. Studies were included if they: (1) focused on adolescents (13–18 years), (2) evaluated suicide or self-harm interventions using digital technologies or (3) reported quantitative outcomes related to suicidal ideation or behaviour.

As for the exclusion criteria, studies were excluded if they: (1) did not include adolescents as the primary population, (2) were reviews, editorials or conference abstracts, (3) only presented the protocol, (4) were not interventions with technological devices or (5) were not fully available (full text).

**Fig. 1.**
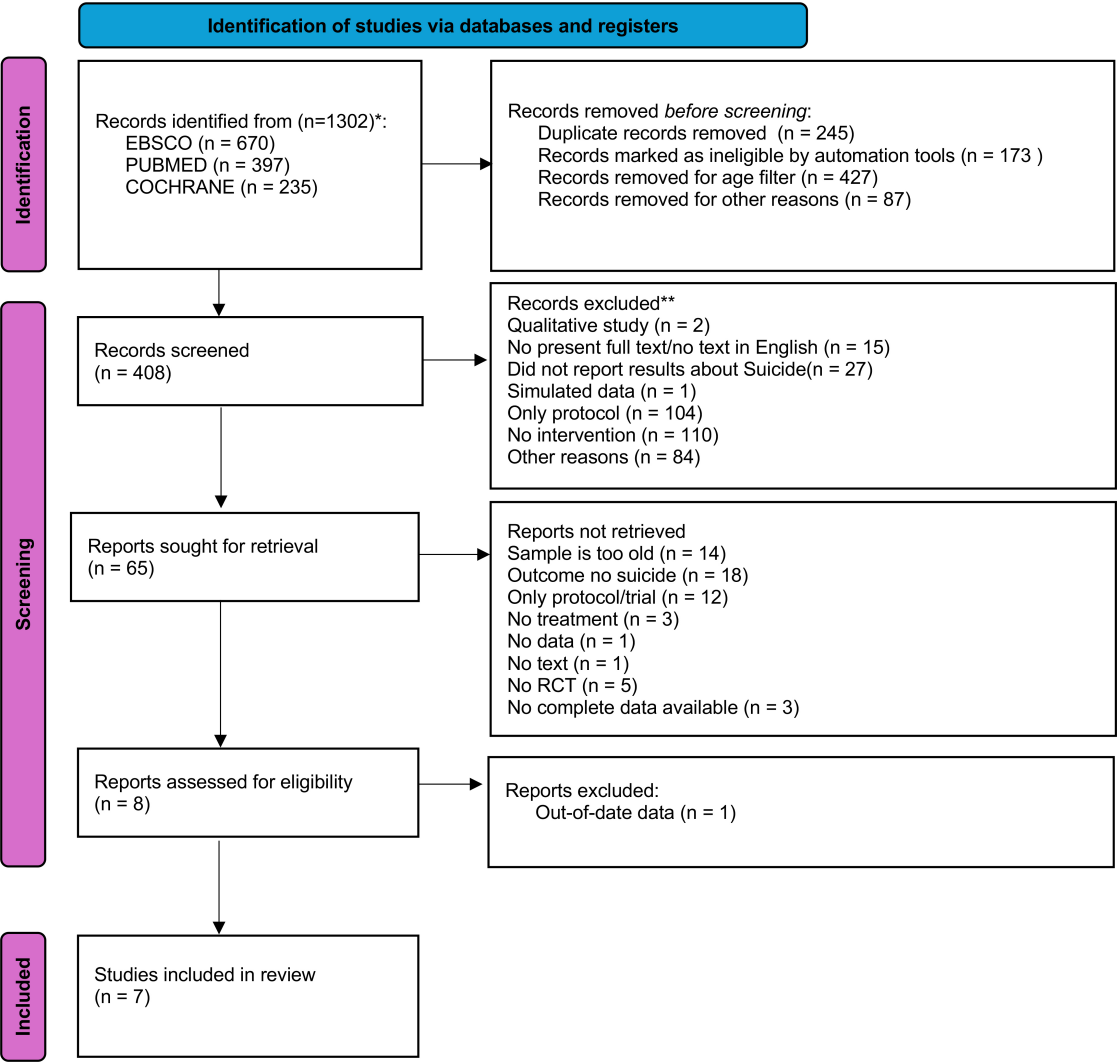
**Flow diagram according to the PRISMA model for systematic review**. PRISMA, Preferred Reporting Items for Systematic reviews and Meta-Analyses.

After cleaning duplicates and ineligible studies for the reference tools, two independent reviewers (M.M. and F.C.) screened and selected the articles. Disagreements were resolved through discussion or consultation with a third reviewer (M.A.C.). Fig. 1 synthesises the entire process along the PRISMA flowchart.

The search strategy intentionally included broad terms like “suicidal risk”, “self-harm” and “self-injury” to maximise sensitivity, as self-harm and non-suicidal self-injury (NSSI) are well-established predictors of subsequent suicidal ideation and behaviours in adolescents. However, during study selection and data extraction, only studies that reported quantitative measures of suicidal ideation were eligible for inclusion in the meta-analytic synthesis, as this was the only suicidal outcome consistently available across the studies.

### Data Analysis

A total of 6 studies were considered in the final sample suitable for review and, in turn, in the meta-analysis, as they met every selected criterion for inclusion. Given that two studies in the sample referred to the same cohort [19, 20], the selection of the effect size was based on the temporal homogeneity of the follow-up periods relative to baseline. As all other included studies reported follow-ups shorter than one year, the one-year follow-up study by Mehlum* et al*. [20] was excluded from the statistical analyses. The main characteristics of each study are summarised in Table 1 (Ref. [19, 21, 22, 23, 24, 25, 26]).

**Table 1.** **Summary of articles included in the systematic review**.

Authors	Trial registration code	Type of study	N intervention	N control	Follow-up duration	Type of intervention	Type of control	Effect size (std. error)	Main results
Czyz E.K.* et al*. [21]	ClinicalTrials.gov	RCT	40	40	1 month	Phone call-based	Self-administered intervention	0.17 (0.22)	Participants of the intervention group (with booster calls) did not report a significant reduction in suicidal ideation.
	NCT03838198								
Dobias M.L.* et al*. [22]	ClinicalTrials.gov NCT04498143 + OSF	RCT	286	279	3 months	Web platform-based	Self-administered web platform	–0.10 (0.08)	Levels of suicidal ideation did not significantly differ between intervention and control group.
Gaete J.* et al*. [23]	Published protocol [24]	RCT	33	20	3 months	Web platform-based	TAU	0.54 (0.29)	Suicidal ideation levels in intervention group declined significantly relative to the control group.
Hetrick S.E.* et al*. [25]	ACTRN12613000864729	RCT	26	24	22 weeks	Web platform-based	TAU	0.33 (0.28)	﻿Even if there was a larger decrease in suicidal ideation in the intervention group relative to the control group, it was not statistically significant.
Mehlum L.* et al*. [19]	ClinicalTrials.gov NCT00675129	RCT	39	38	19 weeks	Phone call-based	No phone calls	0.75 (0.23)	Levels of suicidal ideation declined significantly relative to the control group.
Nuñez D.* et al*. [26]	ClinicalTrials.gov NCT05229302	RCT	51	49	1–5 months	Web platform-based	TAU	0.48 (0.21)	Significant decline in suicidal ideation for the intervention group relative to the control group.

*Note:* RCT, Randomised control trial; TAU, Treatment as usual.

The JASP software (version 0.95.3; JASP Team, 2025, Amsterdam, The Netherlands) was employed for all statistical analyses, namely the meta-analysis module. The total sample size with all the studies was 475 for the intervention samples and 450 for the control samples. Three different models were developed for the meta-analysis. The first, a random effects model, served to assess the overall effect size of the studies. The choice of a random effects model was justified by the fact that there were no reasons to think that all the studies were functionally identical and conducted in the same way [27]. The second and third models incorporated moderator variables by conducting meta-regression analyses [28]. More specifically, the type of intervention (defined by the specific technology used) and the type of control condition (whether the control was exposed to treatment as usual [TAU] or other forms) were considered. These moderators were selected not only based on their consistent availability across all considered studies but also because prior literature had highlighted how these variables can influence studies’ general effects [29]. The effect size was computed as Hedge’s *g*, which represents a more accurate estimate when considering small samples, such as that considered in this study [30]. The considered indexes were standardised mean difference (SMD), heterogeneity indexes (I^2^, τ^2^) and the coefficients of the considered moderators in the different models. Table 2 synthesises the main outputs of each model. A risk bias assessment of the chosen studies was conducted, adapting Cochrane’s guidelines for the assessment of bias risk, namely Risk of Bias 2 (RoB2) [31], and the results are presented in Table 3 (Ref. [19, 21, 22, 23, 25, 26]).

**Table 2.** **Synthesis of model results**.

Model	SMD	*p*	I^2^
Model 1	0.32	0.03	68.02
Model 2	0.33	0.08	68.00
Model 3	0.36	0.03	21.45

*Note:* SMD, standardised mean difference.

**Table 3.** **Analysis of bias risk in the studies**.

Study	D1: Randomisation process	D2: Deviation from the intended interventions	D3: Missing outcome data	D4: Measurement of outcome	D5: Selection of reported results	Risk of bias
Czyz E.K.* et al*. [21]	Low	Some concerns	Low	Low	Low	Low
Dobias M.L.* et al*. [22]	Low	Low	High	Some concerns	Low	High
Gaete J.* et al*. [23]	Some concerns	Some concerns	Low	Some concerns	Low	Moderate
Hetrick S.E.* et al*. [25]	Low	Some concerns	High	Some concerns	Low	High
Mehlum L.* et al*. [19]	Low	Some concerns	Low	Low	Low	Low
Nuñez D.* et al*. [26]	Some concerns	Some concerns	Low	Some concerns	Low	Moderate

## Results

The results of each model—effect size, model coefficients and the lower and upper limits of the 95% confidence interval—are synthesised below. Although the included interventions differed in format, synchronicity and intensity, they shared a defining feature: the use of digital communication technology as a primary vehicle through which to deliver therapeutic content targeting suicidal ideation (plans, attempts, non-fatal behaviours and self-harm/injury were not found in this review). This conceptual commonality justified grouping them under the umbrella of TIs.

**Fig. 2.**
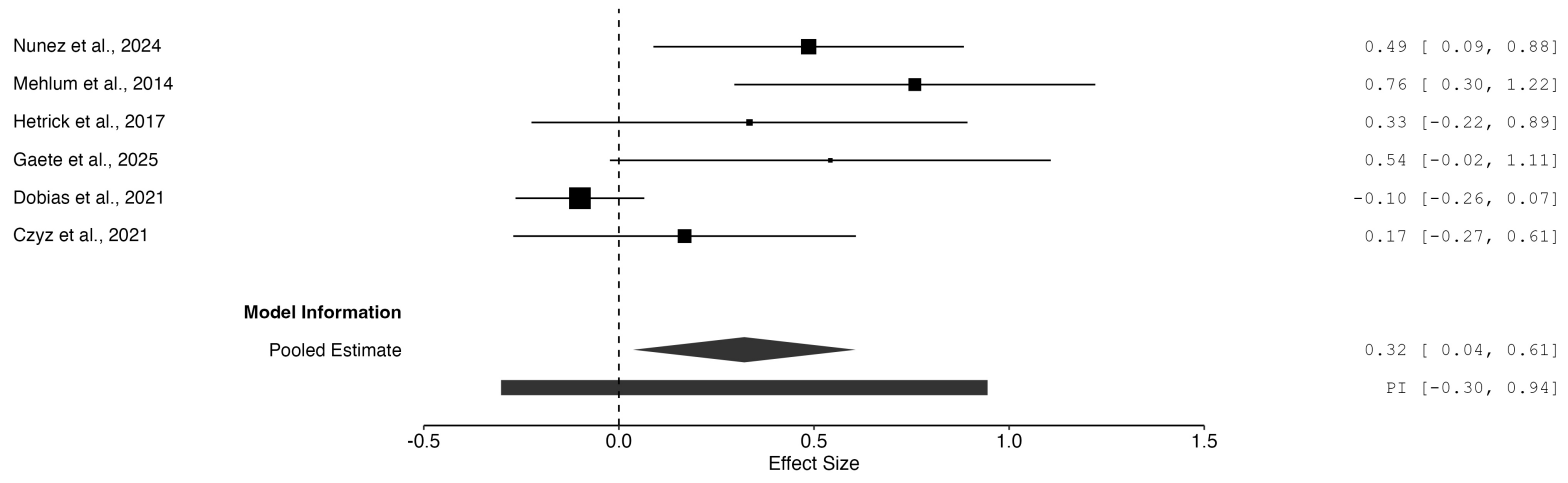
**Forest plot**.

The results related to each study are reported in a forest plot in Fig. 2. (Ref. [19, 21, 22, 23, 25, 26]).

### Model 1: Overall Effect Size

The random-effects meta-analysis indicated a statistically significant moderate overall effect of TIs in reducing suicidal ideation among adolescents (SMD = 0.321, *p* = 0.03, 95% CI [0.036, 0.607]). This suggests that, on average, participants on the receiving end of TIs experienced greater reductions in suicidal ideation than those in the control groups. However, there was substantial between-study heterogeneity, as indicated by the overall test (Q (5) = 20.13, *p* < 0.01, I^2^ = 68.02%), suggesting that nearly 70% of the observed variability in effect sizes is due to real differences between studies. The 95% prediction interval ranged from –0.302 to 0.945, reflecting considerable uncertainty in the expected effects in future studies, perhaps due to the inclusion of studies with different formats, populations and control conditions.

### Model 2: Adding Moderator Variable: “Type of Intervention”

As previously described, to explore potential sources of the heterogeneity seen in the first model, a meta-regression was conducted with the type of intervention (web platform vs. phone call) as a moderator. The model yielded a statistically insignificant pooled effect size (SMD = 0.327, *p *= 0.08, 95% CI [–0.063, 0.717]). Even when a moderator variable was added, heterogeneity persisted at a moderate level (I^2^ = 68%, τ^2^ = 0.088), and the overall moderation test was not significant (F(1, 4) = 0.42, *p* = 0.55), indicating that intervention type did not explain a substantial proportion of the variance. The coefficient for web platform-based interventions, relative to the reference category (phone calls), was negative and statistically insignificant (b = –0.196, *p* = 0.55, 95% CI [–1.031, 0.640]), indicating no differences in effectiveness between the two intervention types. Two of the studies in the web platform group were rated as high-risk, which may partially account for this trend, although the limited sample size prevents any definitive conclusions.

### Model 3: Adding Moderator Variable: “Type of Control Group”

In the second meta-regression model, the type of delivered control was considered (TAU, web-based self-guided control, no phone calls). For this model, the pooled effect size remained statistically significant (SMD = 0.364, *p* = 0.03, 95% CI [0.042, 0.685]), and the residual heterogeneity decreased considerably (Q (3) = 3.57, *p* = 0.31), with I^2^ = 21.45%, indicating that this moderator accounted for a meaningful proportion of between-study variance. The web-based self-guided control group exhibited no significant differences in intervention effects relative to the no-calls control groups (b = –0.552, *p* = 0.09, 95% CI [–1.287, –0.184]). Similarly, TAU did not differ significantly from the no-calls control groups (b = 0.008, *p* = 0.97).

The reduction in I^2^ when introducing control group type as a moderator suggests that the nature of the comparator condition may contribute to the observed heterogeneity. However, both the fact that the web-based self-guided control and TAU did not exhibit significant differences in intervention effects and the fact that the omnibus test of moderation appeared statistically insignificant (F (2, 3) = 4.36, *p* = 0.13) suggest that these results should be interpreted with caution.

**Fig. 3.**
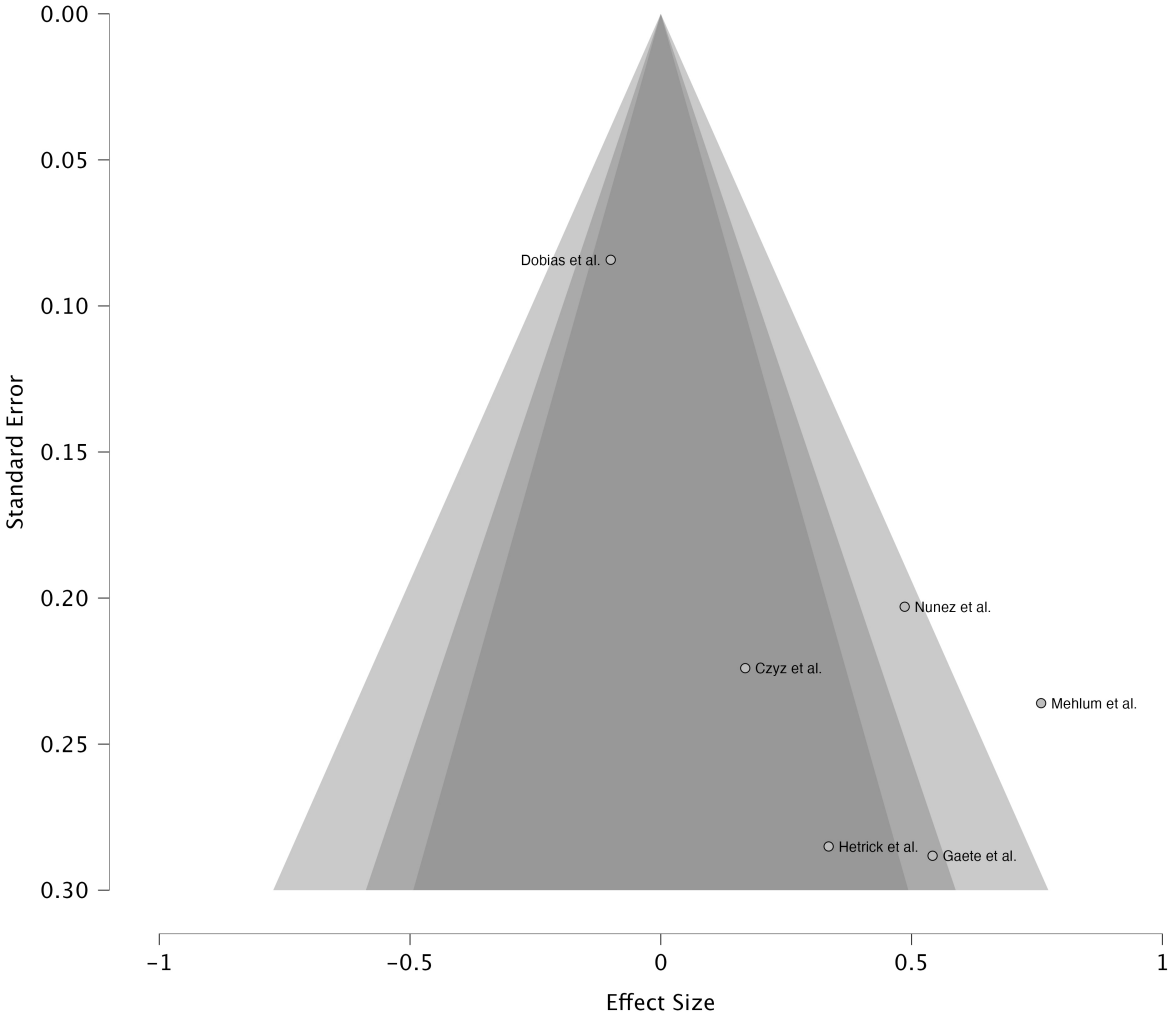
**Funnel plot**.

Given the small number of considered studies (*k* = 6), the statistical power to detect asymmetry is low, and formal publication bias tests (e.g., Egger’s regression test) may be unreliable [32]. Nevertheless, Egger’s test was conducted and revealed statistical significance, highlighting the presence of asymmetry (z = 3.32, *p* < 0.001). Moreover, an inspection of the funnel plot (Fig. 3) highlights an asymmetry in the distribution of the studies. More specifically, only one study appears in the left area, indicating potential publication bias. However, these results should be considered in light of the previously described conditions, particularly the small sample size of the meta-analysis.

### Analysis of the Studies’ Bias Risk 

Additionally, in line with Cochrane’s guideline for the assessment of bias risk (RoB2) [31], the study’s key features were considered and evaluated: sample randomisation, deviation from the intended interventions, missing outcome data and the measurement of the outcomes and biases pertaining to the selection of reported results. For each study, these features were evaluated and combined to obtain an overall bias risk (from “low” to “high”). The summary of the assessment of bias risk is reported below in Table 3. The risk of bias was divided equally within the sample: two studies were assessed as low-risk, two as moderate-risk, and two as high-risk.

The assessment of bias risk revealed heterogeneous methodological quality among the considered studies. High-risk studies presented some critical issues related to the high dropout rate at follow-up, while moderate-risk studies showed more problems with recruitment biases. The assessment of bias risk, alongside the previously reported findings, suggests that the results of the meta-analysis should be considered with caution due to the impact of these study characteristics on the overall outcome.

## Discussion

The objective of this meta-analysis was to examine the effectiveness of TIs for suicide prevention among adolescents. Although its conceptual focus was on the broader construct of suicidal risk—ideation, planning, behaviour and self-harm—the evidence base for adolescent TIs is largely limited to suicidal ideation outcomes. Consequently, suicidal ideation represents the only outcome that is consistently available for quantitative synthesis. The findings indicate that TIs yield an overall statistically significant moderate reduction in suicidal ideation, aligning with other studies that have highlighted the positive effect of technology-assisted treatments on adolescents’ mental health [33, 34].

The observed effects, without accounting for moderating variables, were accompanied by a high level of cross-study heterogeneity, reducing the clarity and significance of the findings. In contrast, models that included moderators exhibited lower heterogeneity, allowing for a more coherent interpretation of the results. More precisely, the analysis of the type of technology used in the interventions yielded non-significant results, indicating that the interventions’ effectiveness does not vary substantially based on the employed technological format [35].

Mohr* et al*. [6] suggest that these interventions, known as behavioural intervention technologies, may be delivered through various channels without losing their fidelity or clinical effectiveness. The lack of significant differences between phone call- and web platform-based interventions on adolescent suicidal ideation may be attributed to the comparable quality of interaction and the functional equivalence of these technological modalities. This includes aspects like therapeutic alliance, empathy, attentiveness, and the patient’s willingness to disclose information [36]. Additionally, components tailored to the adolescent’s needs are likely equivalent across both modalities. Another factor could be the standardisation of protocols, which ensures that delivery methods (e.g., phone, web) do not significantly impact outcomes [37].

The consideration of different types of control groups yielded more encouraging results, with greater effects observed when the control group was exposed to technology compared to those receiving TAU. These findings suggest that the effectiveness of the intervention may be influenced by the degree of disparity between the intervention and control conditions—particularly when the control group is not exposed to any form of technological support. These results align with those of Grist* et al*. [29], who found that technology-based interventions for depression and anxiety among adolescents exerted different effects based on the control group, with a small effect size when the control group was a placebo and a moderate effect size when the control group was a wait-list group. Thus, generally speaking, the results obtained through this meta-analysis align with those in the broader literature on adolescents’ mental health issues [5, 38].

Even if the obtained results are promising, this study has several limitations that must be noted here. First, the number of included studies was small (*k* = 6), limiting statistical power, particularly for detecting publication bias or drawing robust moderator conclusions. Given the limited statistical power of the analysis, this work should be considered exploratory in nature. Consequently, the reported findings do not provide definitive conclusions; rather, they offer a preliminary overview of the state of the art, not allowing for the generalisation of the results to the broader population of adolescents. The small sample size also limited the exploration for publication bias, even if we categorised each study by level of bias risk and found that only two studies exhibited a high risk of bias based on the criteria (as reported in Results section). The sample size further limited our ability to conduct a subgroup analysis, as this methodology has a high risk of reporting unreliable results [39]. Moreover, actual suicidal attempts and self-harm events were rarely reported across the considered studies and, therefore, could not be synthesised. As a result, the conclusions of this review are strictly limited to suicidal ideation. One key limitation of this review is the substantial heterogeneity across intervention formats. Phone-delivered therapeutic contacts and web platform-based interventions differ in their dose, intensity and therapeutic process. Although these modalities can be conceptualised as different iterations of BITs, their variability likely contributes to the observed heterogeneity in effect sizes, limiting the strength of modality-related conclusions. In fact, it is not clear how different platforms influence levels of suicidal ideation, considering also that some platforms require more interaction with technology than others (e.g., web platforms vs. phone calls). Furthermore, the studies considered in this review only reported short-term effects, as only one of the studies was a follow-up on a treatment. This could also be due to the recent rise in interest in adopting this type of platform in suicidal ideation interventions.

The quality of the studies included in this meta-analysis was assessed in line with Cochrane’s guidelines for the assessment of bias risk (RoB 2) [31]. Key methodological features, such as sample randomisation, deviation from the intended interventions, missing outcome data and the measurement of the outcomes and biases pertaining to the selection of reported results. As reported above, the heterogeneity of bias risk across the studies represents an important element, pointing to a need for cautious interpretation of the findings. The Egger’s test and the inspection of the funnel plot suggested a high probability of publication bias [40], highlighting the need to interpret results with caution. Future research should aim to minimise these biases in order to enhance the reliability of the results.

## Conclusions

This systematic review and meta-analysis provides preliminary evidence that technology-assisted interventions can effectively reduce suicidal ideation among adolescents. The findings highlight the potential of digital approaches as accessible and engaging tools for suicide prevention in a population that is highly connected to technology [12]. Even with its limitations, the results of this meta-analysis offer some important insights into the field of TIs for adolescents. The implementation of TIs represents a promising strategy with which to treat their mental health, as they may be particularly suitable for them. The fact that the number of identified studies is relatively small also highlights the need to further explore this stream of research by investigating how this type of intervention can improve intervention feasibility and the participation of adolescents. Certainly, further RCT studies are necessary to understand the actual mechanism that influences this type of intervention’s effects on suicidal ideation.

## Availability of Data and Materials

The data that support the findings of this study are available from the corresponding author upon reasonable request.
